# Soluble Receptor for Advanced Glycation End Products: A Protective Molecule against Intramyocardial Lipid Accumulation in Obese Zucker Rats?

**DOI:** 10.1155/2019/2712376

**Published:** 2019-02-28

**Authors:** Elena Dozio, Elena Vianello, Francesco Bandera, Erika Longhi, Stefano Brizzola, Manuela Nebuloni, Massimiliano M. Corsi Romanelli

**Affiliations:** ^1^Department of Biomedical Sciences for Health, Università degli Studi di Milano, Milan, Italy; ^2^Department of Cardiology, I.R.C.C.S. Policlinico San Donato, San Donato Milanese, Milan, Italy; ^3^Department of Biomedical and Clinical Sciences “Luigi Sacco”, Università degli Studi di Milano, Milan, Italy; ^4^Department of Veterinary Medicine, Università degli Studi di Milano, Milan, Italy; ^5^Service of Laboratory Medicine-Clinical Pathology, I.R.C.C.S. Policlinico San Donato, San Donato Milanese, Milan, Italy

## Abstract

Most of the obesity-related complications are due to ectopic fat accumulation. Recently, the activation of the cell-surface receptor for advanced glycation end products (RAGE) has been associated with lipid accumulation in different organs. Nevertheless, the role of RAGE and sRAGE, the soluble form that prevents ligands to activate RAGE, in intramyocardial lipid accumulation is presently unknown. To this aim, we analyzed whether, in obesity, intramyocardial lipid accumulation and lipid metabolism-related transcriptome are related to RAGE and sRAGE. Heart and serum samples were collected from 10 lean (L) and 10 obese (OB) Zucker rats. Oil red staining was used to detect lipids on frozen heart sections. The lipid metabolism-related transcriptome (84 genes) was analyzed by a specific PCR array. Heart RAGE expression was explored by real-time RT-PCR and Western blot analyses. Serum levels of sRAGE (total and endogenous secretory form (esRAGE)) were quantified by ELISA. Genes promoting fatty acid transport, activation, and oxidation in mitochondria/peroxisomes were upregulated in OB hearts. Intramyocardial lipid content did not differ between OB and L rats, as well as RAGE expression. A slight increase in epicardial adipose tissue was observed in OB hearts. Total sRAGE and esRAGE concentrations were significantly higher in OB rats. sRAGE may protect against obesity-induced intramyocardial lipid accumulation by preventing RAGE hyperexpression, therefore allowing lipids to be metabolized. EAT also played a protective role by working as a buffering system that protects the myocardium against exposure to excessively high levels of fatty acids. These observations reinforce the potential role of RAGE pathway as an interesting therapeutic target for obesity-related complications, at least at the cardiovascular level.

## 1. Introduction

Obesity is one of the leading risk factors for cardiovascular diseases [1]. Most of the obesity-related complications may deal with fat accumulation in tissues different from the adipose one, among which are the liver, muscle, and pancreas [2–5]. This can take place also in the heart where lipid deposition may promote organ damage and dysfunction by inducing abnormalities in cardiac cell metabolism as well as structural adaptation of the cardiovascular system [6]. Intramyocardial lipid accumulation has been observed in different animal models of obesity [7, 8]. Human studies also demonstrated an existing association between myocardial fat content and adiposity [9–12]. Although preclinical studies described some potential cellular and molecular mechanisms linking obesity to heart steatosis [13–16], the identification of additional pathways and potential targets that could be useful to prevent and/or reverse the detrimental effects of obesity at the cardiovascular level is a compelling need.

Recent insights, also from our group, demonstrated the involvement of the cell membrane receptor for advanced glycation end products (receptor for AGEs (RAGE)), a known trigger of inflammation and oxidative stress [17–19], in inducing adipocyte hypertrophy, adipose tissue expansion, and also ectopic lipid accumulation in different organs, such as the liver [20–24]. Contrarily, its corresponding soluble form, sRAGE, seems to work as a decoy receptor. By binding RAGE ligands in the circulation, sRAGE can prevent membrane RAGE activation and related detrimental effects. Among the different forms that compose the circulating sRAGE pool, namely, cRAGE and esRAGE, the former is the most abundant, but the real decoy receptor seems to be the latter. The circulating levels of total sRAGE and the different forms have also been suggested as biomarkers of different cardiometabolic complications [25–28].

Nevertheless, the role of RAGE and sRAGE in heart steatosis is presently unknown. In this study, we aimed to analyze whether, in obesity, intramyocardial lipid accumulation and lipid metabolism-related transcriptome are related to RAGE and sRAGE forms by using Zucker rats as a model of obesity.

## 2. Materials and Methods

### 2.1. Animal Model and Tissue Collection

Ten obese nondiabetic male Zucker rats (OB) (fa/fa, 10 weeks of age) and 10 lean littermates (L) (Fa/?) were purchased from Charles River Laboratories (Calco, Lecco, Italy). The rats were housed at constant room temperature (22 ± 2°C) and humidity (60 ± 5%) with a light-dark cycle of 12 hours each and fed a standard rodent chow (10% fat) and water ad libitum. At the age of 25 weeks, the rats were anesthetized with zoletil (20 mg/kg) and sacrificed by cervical dislocation. Ten hearts (five L and five OB) were stored in Allprotect Tissue Reagent (QIAGEN, Hilden, Germany) at -20°C until RNA and protein extraction. The remaining hearts were fresh frozen in OCT for cryosectioning. Blood was obtained by cardiac puncture, and after cloating, serum was isolated by centrifugation at 1500 g for 15 min. The Italian Ministry of Health approved the procedures of animal care, anesthesia, euthanasia, and tissue collections for this study (Ministerial Authorization 325/2015PR of 2015/04/05).

### 2.2. Heart Lipid Staining

Staining of lipids was performed with Oil Red O (ORO) dye (Sigma-Aldrich, Milan, Italy). Briefly, 10 *μ*m cryostat sections were air dried, formalin fixed (5 minutes in 10% ice-cold formalin), and washed with running tap water. After rinsing with 60% isopropanol, the sections were stained with freshly prepared ORO working solution, obtained by diluting ORO with deionized water in a ratio of 3 : 2, for 15 minutes. A second wash with 60% isopropanol and a counterstaining with hematoxylin were performed. A coverslip was applied by using an aqueous medium. The lipid resulted in a red stain, while the nuclei in blue. Slides were visualized with a Nikon Eclipse 80i microscope, and images were captured with the attached digital camera and image acquisition software.

### 2.3. RNA Extraction and Reverse Transcription

Total RNA was isolated from rat hearts using the RNeasy Lipid Tissue Mini Kit (QIAGEN), according to the manufacturer's instructions. Elution was performed with 30 *μ*L of RNase-free water, and the RNA concentration was quantified with NanoDrop (Thermo Fisher Scientific, Waltham, MA). RNA samples (1 *μ*g) were first treated with a genomic DNA elimination step (42°C for 5 min) and then reversely transcribed in 20 *μ*L using the RT^2^ First Strand Kit according to the manufacturer's instructions (QIAGEN).

### 2.4. Real-Time PCR Assay

The lipid metabolism-related transcriptome was evaluated by real-time PCR using the ready-to-use rat *Fatty Acid Metabolism* RT^2^ Profiler PCR Array (PARN-007Z, QIAGEN) which profiles the expression of 84 key gene transcripts involved in lipid metabolism. Each cDNA sample was diluted with nuclease-free water and mixed with the RT^2^ SYBR green Mastermix (QIAGEN). Twenty-five *μ*L of the experimental mixture was added to each well of the rat *Fatty Acid Metabolism* array (one array for each cDNA). Real-time PCR was performed on the CFX96 thermocycler (Bio-Rad, Milan, Italy) using the following cycling conditions: (1) 95°C/10 min and (2) 40 cycles: 95°C/15 sec followed by 60°C/1 min. Dissociation curves were then performed to verify PCR specificity using the default melting curve program of the instrument. Data were analyzed using the QIAGEN RT^2^ Profiler PCR Array Data Analysis Web Portal. RAGE gene expression was carried out according to the manufacturer's instructions using specific rat primers (PPR44508A, QIAGEN) and the same mastermix, thermocycler, and conditions previously indicated. Each sample was run in triplicate, and the fold difference between groups was evaluated by the ΔΔCt method.

### 2.5. Analysis of Real-Time PCR Array

Each array contained 5 housekeeping genes for normalization, 1 genomic DNA control, 3 reverse transcription controls, and 3 positive PCR controls. Following real-time PCR, data from all the arrays were analyzed using the same threshold. Cycle threshold (Ct) values > 35 were considered a negative call. Ct for genomic DNA controls > 35 and Ct of 20 ± 2 for the positive PCR controls confirmed the lack of DNA contamination and efficient PCR amplification, respectively. According to the manufacturer's protocol, normalization of the expression data of the analyzed samples can be performed using one of the housekeeping genes or any other of the 84 genes, provided that the Ct values of the gene used for normalization does not differ more than 1.5 cycles in all samples (plates). Among the housekeeping and tested genes, SLC27A4 was identified as the most stable gene between our groups and therefore selected as the housekeeping gene for our study. Normalization was performed by calculating the ΔCt for each gene in the plate. The RT^2^ Profiler PCR Array Data Analysis Web Portal was used to calculate the fold change based on the ΔΔCt method. For fold-change values greater than 1, the results were reported as fold upregulation. For fold-change values less than 1, the negative inverse of the results are reported as fold downregulation. The *p* values were calculated using the QIAGEN RT^2^ Profiler PCR Array Data Analysis Web Portal and were based on Student's *t*-test (two-tail distribution and equal variances between the two samples) on the replicate 2-ΔCt values for each gene in each treatment group compared to the control group and considered significant for values < 0.05.

### 2.6. Western Blot Analysis

Samples of heart tissues were homogenized with TissueLyser II (QIAGEN) in ice-cold RIPA lysis buffer (50 mM Tris-HCl, pH 7.5, 150 mM NaCl, 1 mM EDTA, 1 mM EGTA, 1% Triton X-100, 0.1% SDS, 0.5% Na-deoxycholate, and 50 mM sodium fluoride) containing 1% protease inhibitor cocktail (Sigma-Aldrich, Milan, Italy). The homogenate was kept on ice for 30 min, centrifuged at 500 rpm for 10 min at 4°C, and the resulting supernatant centrifuged at 13,200 rpm for 15 min at 4°C. Protein concentration was determined with the Quantum Protein Assay Kit, based on the BCA reagent (EuroClone, Milan, Italy). Equal amounts of protein samples (50 *μ*g) were suspended in Laemmli sample buffer and separated using 4-20% Mini-PROTEAN TGX Stain-Free Gels and Tris/Glycine/SDS running buffer (Bio-Rad). After SDS-electrophoresis, TGX Stain-Free Gels were activated for 1 min and imaged using the ChemiDoc Touch System and the Image Lab 5.2.1 software (Bio-Rad). The separated proteins were then transferred from the gel to a nitrocellulose membrane using the Trans-Blot Turbo Mini Nitrocellulose Transfer Packs and the Trans-Blot Transfer System (Bio-Rad). The membranes were blocked with 5% dry milk in Tris-buffered saline/0.1% Tween 20 for 1 h at room temperature, and the blots were then incubated overnight at 4°C with a diluted solution of the primary anti-RAGE antibody (1 *μ*g/mL) (Abcam, Cambridge, UK). The subsequent incubation with a secondary antibody conjugated with peroxidase was performed at room temperature for 2 h. Immunoreactivity was detected by a working solution (Clarity Western ECL Substrate, Bio-Rad) and the ChemiDoc Touch System. Analysis included the determination of total stain-free fluorescence and signal for RAGE of each sample/lane on the blots using the Image Lab software. RAGE signals were normalized with stain-free total lane volumes [29].

### 2.7. sRAGE and esRAGE Enzyme-Linked Immunosorbent Assays (ELISA)

Circulating levels of sRAGE and esRAGE were quantified on serum samples according to the manufacturer's directions, with the following assays: rat sRAGE duo set ELISA (DY1616, R&D System, Minneapolis, MN) and rat esRAGE ELISA (E-EL-R2497, Elabscience, Houston, TX). The GloMax®-Multi Microplate Multimode Reader was used for photometric measurements (Promega, Milan, Italy).

### 2.8. Statistical Analysis

Data are expressed as mean ± SD. The normality of data distribution was assessed with the Kolmogorov-Smirnoff test. *t*-test or Mann-Whitney tests were used for group comparisons. Data were analyzed using the GraphPad Prism 5.0 biochemical statistical package (GraphPad Software, San Diego, CA). A *p* value < 0.05 was considered significant.

## 3. Results

### 3.1. Fat Staining on Heart Tissues

**Figure 1:**
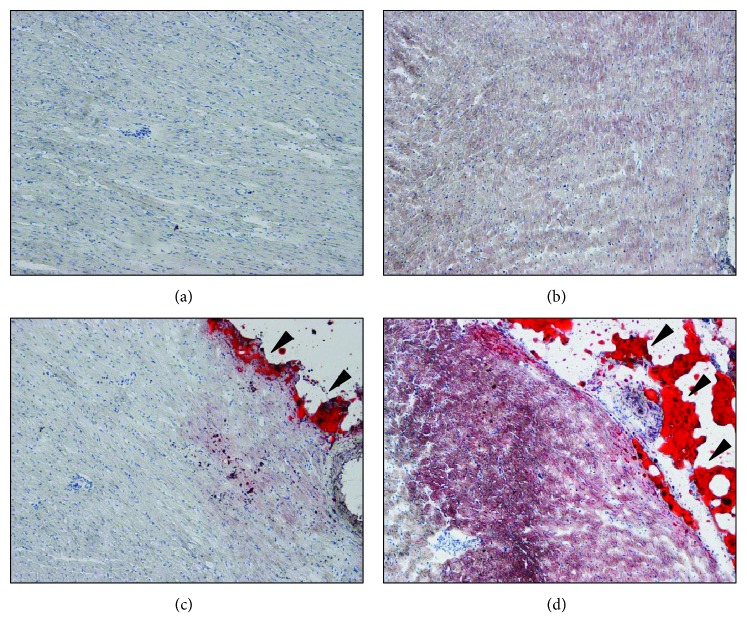
Fat staining on heart tissues. Ten *μ*m heart cryostat sections were stained with Oil Red O dye. (a) and (c) are representative pictures of lean heart tissues and (b) and (d) of obese heart tissue. Red staining in (c) and (d) indicates (arrowheads) epicardial adipose tissue. Images were all captured at 10x magnification.

In the myocardium, no lipid accumulation was found, either in the L (Figure 1(a)) or in the OB rats (Figure 1(b)). In both the L and OB animals, a small amount of fat (red staining) was visible in the atrioventricular groove underneath the epicardium (epicardial adipose tissue (EAT)). Compared to the L animals (Figure 1(c)), the accumulation was slightly more consistent in the OB rats (Figure 1(d)).

### 3.2. Evaluation of Heart RAGE Expression

**Figure 2:**
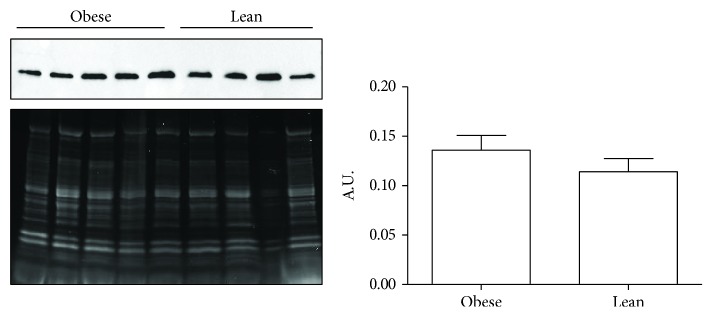
Western blot analysis of RAGE expression in obese and lean hearts. RAGE protein levels were quantified in 5 obese and 4 lean heart samples by Western blot analysis. The fifty *μ*g protein extract/lane was analyzed with the anti-RAGE antibody. A representative blot, the corresponding stain-free gel utilized for protein normalization, and the semiquantitative analysis are shown. Data are expressed as mean ± SD.

We compared OB and L hearts in term of RAGE expression. RAGE did not differ between the two animal groups at both the gene (fold change: -1.3, *p* > 0.05) and the protein level (Figure 2, *p* > 0.05).

### 3.3. Evaluation of Lipid Metabolism-Related Transcriptome in Hearts

**Figure 3:**
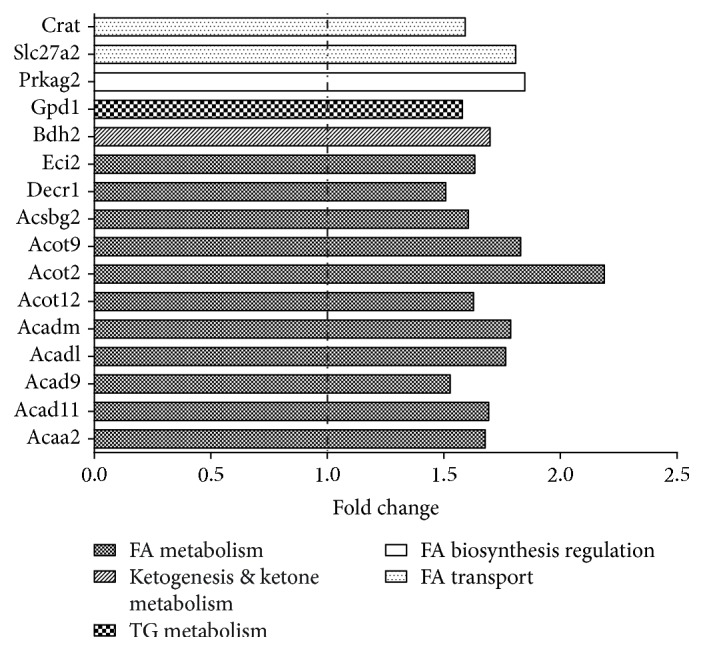
Lipid metabolism-related transcriptome in obese and lean hearts. Gene expression profile was evaluated using the rat *Fatty Acid Metabolism* RT^2^ Profiler PCR Array. Genes that showed a fold change > 1.5 in obese vs. lean groups are represented and grouped according to their biological function.

The rat *Fatty Acid Metabolism* RT^2^ Profiler PCR Array allowed us to assess the expression of 84 genes involved in fatty acid metabolism, fatty acid transport, fatty acid biosynthesis regulation, ketogenesis and ketone body metabolism, and triacylglycerol metabolism. Among the 84 genes, 16 had a Ct between 30 and 35 in both OB and L rats, which means low levels of expression, and 68 had a Ct below 30. As shown in Figure 3, 16 genes were upregulated in OB hearts. Among these, 9 genes are involved in fatty acid metabolism. In detail, one (*Acaa2*) is an acetyl-CoA transferase, 4 (*Acad11*, *Acad9*, *Acadl*, and *Acadm*) are acetyl-CoA dehydrogenases, 3 (*Acot12*, *Acot2*, and *ACot9*) are acetyl-CoA thioesterases, and 1 (*Acsbg2*) is an acetyl-CoA synthetase. One gene (*Bdh2*) is involved in ketogenesis and ketone body metabolism, 1 (*Gpd1*) is involved in triacylglycerol metabolism, 1 (*Prkag2*) participates in the regulation of fatty acid biosynthesis, and 2 are involved in fatty acid transport (*Crat* and *Slc27a2*). The other two genes (*Decr*1 and *Eci2*) are also involved in metabolic pathways that regulate fatty acid metabolism. A statistically significant difference was also observed for two of these genes (*Prkag2* and *Slc27a2*) (*p* value < 0.05, OB vs. L).

### 3.4. Evaluation of sRAGE and esRAGE Plasma Levels

**Figure 4:**
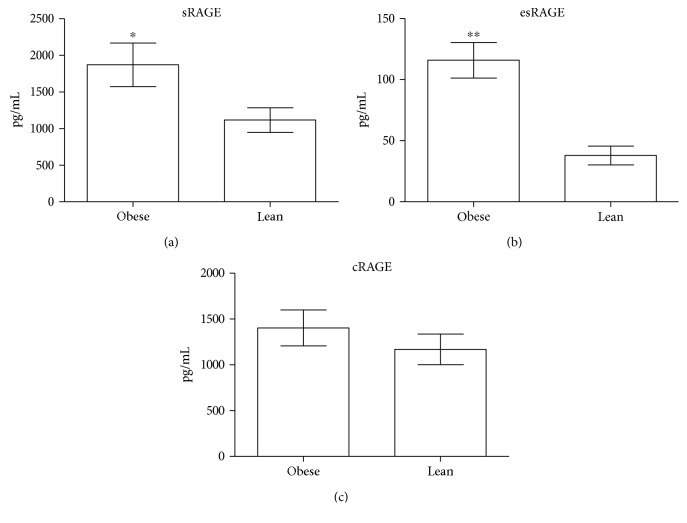
Serum levels of sRAGE, esRAGE, and cRAGE in obese and lean rats. Serum levels of total sRAGE (a) and esRAGE (b), quantified in serum samples, were higher in obese than in lean animals. Levels of cRAGE were calculated as the difference between the total and the esRAGE form and did not differ between the two groups (c). Data are expressed as mean ± SD. ^∗^*p* < 0.05 and ^∗∗^*p* < 0.001 vs. the lean group.

Circulating levels of total sRAGE and esRAGE were quantified on serum samples. cRAGE was calculated as the difference between the total and the esRAGE form instead. As shown in Figure 4, sRAGE levels (Figure 4(a)) were higher in OB than in L (1869.00 ± 296.90 vs. 1115.00 ± 166.90 pg/mL, respectively, *p* < 0.05) as well as esRAGE (Figure 4(b)) (115.90 ± 43.85 vs. 37.89 ± 21.67 pg/mL, respectively, *p* < 0.001). Levels of cRAGE (Figure 4(c)) did not differ between the two groups instead (1403.00 ± 588.30 vs. 1169.00 ± 526.80 pg/mL, respectively, *p* = 0.373).

## 4. Discussion

The main finding of this study is the observation that intramyocardial lipid accumulation does not occur in OB rats that display increased circulating levels of sRAGE, namely, the esRAGE form, and that there is no change in the RAGE expression in the heart. The reasons for exploring the association between RAGE, sRAGE, and heart steatosis in obesity were many, but there were two immediate ones: a previously described role of RAGE in promoting lipid accumulation and the lack of information about RAGE and heart steatosis in obesity [21, 22, 30–34].

According to previous findings [35, 36], we expected to observe an increased intramyocardial lipid deposition in OB rats. Differently, fat did not accumulate ectopically in the myocardium, while EAT was slightly increased. Fatty acids are the main fuel for the heart, and intramyocardial fat accumulation may occur when fatty acid availability and oxidation are not properly balanced. In obesity, visceral fat displays an increased level of lipid turnover that, together with an increased release of proinflammatory molecules from adipocytes and/or infiltrating macrophages, gives rise to metabolic complications in different organs [37–39]. The lack of intramyocardial lipid accumulation in OB rats may result from an increased lipid utilization by cardiac cells and storage in EAT. Although the expansion of EAT in obesity led to considering it as a pathological organ, this depot may also play important protective effects on heart metabolism. In fact, EAT has been proposed to provide fatty acids for the myocardium and to function as a buffering system that protects the heart against exposure to excessively high levels of fatty acids [40]. In our obesity model, its slight increase seems thus to play a protective role in terms of heart lipid metabolism. Moreover, from a molecular point of view, data obtained from the lipid metabolism-related transcriptome also suggested the activation of specific metabolic pathways that promote lipid utilization instead of deposition in OB hearts. In particular, the increased expression of *Acaa2* (acetyl-CoA transferase), *Acad11*, *Acad9*, *Acadl* and *Acadm* (acetyl-CoA dehydrogenases), *Acot12*, *Acot2* and *ACot9* (acetyl-CoA thioesterases), *Crat* and *Slc27a2* (fatty acid transporters), *Decr*1 and *Eci2* (accessory enzymes which participate in betaoxidation), and *Acsbg2* (acetyl-CoA synthetase) confirmed the activation of metabolic pathways that, by promoting fatty acid transport, activation, and oxidation in mitochondria/peroxisomes, represent a protective mechanism against fat accumulation. On the other side, the upregulation of *Gpd1*, a key element that connects carbohydrate and lipid metabolism, and *Prkag2*, the noncatalytic subunit of AMP-activated protein kinase that in response to increased intracellular ATP levels activates energy-producing pathways (i.e., biosynthesis), might contribute to increase the synthesis of triacylglycerol. However, the increase in fatty acid oxidation which reduces fatty acids necessary for glycerol 3-phosphate esterification protects against fat accumulation [41].

Which is the role of RAGE in promoting fat accumulation? Previous studies demonstrated a link between increased RAGE expression and activation of pathways that can promote lipid accumulation in many cell types/tissues and suggested that therapies preventing RAGE activation are able to reduce these effects [21, 30, 33, 34, 42]. Results obtained in this study emphasized the potential association between RAGE, lipid accumulation, and genes involved in lipid metabolism in the heart; however, they did not prove a direct cause-effect relationship. Just the manipulation of RAGE expression in *in vitro* studies will be able to confirm the role of the receptor in affecting specific lipid metabolic pathways that can finally lead to lipid accumulation. Another interesting question is why RAGE levels, different from what was expected, did not increase. Obesity is a condition characterized by increased levels of many RAGE ligands, such as AGEs and ALEs (advanced lipoxidation end products). Formation of these products is a naturally occurring process and is the result of normal metabolism. However, their production and accumulation is enhanced under chronic inflammation and oxidative stress, two conditions accompanying obesity, and this may occur before obesity-related complications become manifest. A significant accumulation of RAGE ligands has been described in murine models of obesity, in which these products can promote engagement of the RAGE pathway and RAGE upregulation and detrimentally impact organ function [32, 43–45]. Any mechanism preventing RAGE engagement and activation may thus protect against detrimental effects. sRAGE just plays this protective role, and its upregulation may be acknowledged as a mechanism that prevents RAGE activation and RAGE-mediated ectopic lipid deposition. With regard to sRAGE, it is also important to point out that this soluble form is a pool composed by cRAGE, derived by the proteolytic cleavage of the membrane-bound molecule RAGE, and esRAGE, the endogenous secretory form. Among these forms, an increase in the cRAGE level is considered a surrogate marker of inflammation. In fact, the activation of RAGE and its proinflammatory signaling promotes the expression of membrane RAGE and inflammation-related enzymes, such as the matrix metalloproteinase 9, which upregulate RAGE cleavage and release into the blood [46]. esRAGE, instead, is endogenously secreted. Since it works as a decoy receptor, keeping its levels high is a protective mechanism against RAGE activation, upregulation, and related damaging effects [47–50]. According to our results, the observed increase in esRAGE may be considered a counterregulatory strategy activated by the body to reduce the obesity-related damaging effects. In our animal model, thus, until esRAGE levels are high, no intramyocardial lipid deposition occurs and EAT plays a protective role for the heart too. How long esRAGE levels are kept high and how esRAGE levels potentially protect against heart steatosis are two questions that can be answered by just using animal models of different ages that experience obesity for a longer time and already display different obesity-related cardiometabolic complications. Furthermore, reduction of esRAGE levels may also impact on EAT by promoting a significant enlargement of this depot, as previously described in our human studies [28], and its transformation into pathological organs with a well-documented role in the onset and progression of cardiovascular diseases.

## 5. Conclusions

In conclusion, increased levels of sRAGE, namely, esRAGE, seem to protect against ectopic lipid accumulation in the myocardium by preventing RAGE hyperexpression, promoting fatty acid storage in EAT and their oxidation. These observations reinforce the potential role of RAGE pathway as an interesting therapeutic target for obesity-related complications, at least at the cardiovascular level.

## Data Availability

The data used to support the findings of this study are available from the corresponding author upon request.
